# Current themes in molecular pediatrics: molecular medicine and its applications

**DOI:** 10.1186/1824-7288-36-20

**Published:** 2010-02-19

**Authors:** Andrea Superti-Furga, Livia Garavelli

**Affiliations:** 1 Centre for Pediatrics and Adolescent Medicine, University of Freiburg, Freiburg, Germany; 2 Clinical Genetics Unit, Department of Obstetrics and Pediatrics, Santa Maria Nuova Hospital, Reggio Emilia, Italy

## Abstract

We focus on themes that are derived from clinical practice and research in the field of genetic diseases of bone and inborn errors of metabolism but may be of more general interest as they indicate some trends in molecular medicine as related to pediatrics. Identifying the disease-causing mechanism brings about efficient therapeutic strategies and discovering the mutant genotype in the near future may become helpful for devising custom-built molecular responses. At the same time, the transition of therapy from the experimental phase to industrial application is difficult as there may be novel roles (and potentially conflicting interests) between physicians, patient organisations, governmental agencies and the pharmaceutical industry. Awareness of these potential conflicts may help in recognizing and dealing with these issues.

## Nosology and molecular characterization of genetic disorders

The identification of individual genes responsible for genetic disorders and the characterization of the mutation spectrum and the pathogenetic mechanism have a profound influence on the nosology of diseases. This concept is well illustrated by the group of the so-called skeletal dysplasias and dysostoses. The great heterogeneity in this group of disorders has been first recognized in the Sixties and led to an expanding classification based on clinical and radiographic criteria (with the notable exception of the subgroup of lysosomal storage diseases that were among the first to be characterized biochemically). Unfortunately, the somewhat abstruse denominations of the individual entities and the practical difficulty of diagnosis based on radiographic features made the whole field unfriendly to most pediatricians. Questions pertaining to the legitimacy of the distinction of such a large number of different disorders were raised.

In the Eighties and Nineties, several important genes were identified (COL1A1, COL2A1, FGFR3, DTDST, COMP), each of which was responsible for a whole variety of different disorders. The impression arose that perhaps only a handful of genes with key roles in cartilage and bone would be responsible for the majority of skeletal disorders. This notion is correct in the sense that mutations in these genes are frequent and account for perhaps one half of all instances of skeletal dysplasias.

However, the unrelenting stream of new discoveries shows clearly that the complexity present in the clinical-radiographic classification does indeed have a biological substrate: an unexpectedly large number of genes is involved in skeletal patterning, development and growth, and mutations in these many genes may result in clinically recognizable disease. These genes show great diversity and reflect a variety of molecular mechanisms; the micro-cosmos of genetic skeletal disorders reflects the more general, common themes of genetic mechanisms at large [1-3].

## One gene-one disorder?

One of the most important observations that emerged from the molecular characterization of genetic skeletal diseases is the variety of disorders that can be produced by mutations at a single locus. For example, genes coding for collagen 1, collagen 2, the sulfate transporter, or alkaline phosphatase can all be mutated in many different ways and give rise to phenotypes that range from barely discernible to prenatal lethal. Every single one of these genes is responsible for several "entities" in the clinical-radiographic classification [1,2]. The difference in phenotype can be explained by a gradual loss of function and can thus be positioned on a more or less linear gradient [4,5].

Translation of this concept to other fields, such as that of metabolic diseases, may be instructive. There is no reason to assume that the same degree of variability will not be present in most (if not all) enzyme disorders, and that e.g. methylmalonic acidemia or phenylketonuria would not be similarly heterogeneous. The variability of a disorder secondary to mutational heterogeneity at the responsible locus is a "common theme" in molecular pediatrics.

Some genes produce more than a linear gradient of phenotypes. Thus, collagen 1 genes may result in osteogenesis imperfecta or an Ehlers-Danlos variant according to whether the mutation is in the helical or propeptide domain; filamin A mutations may produce either skeletal malformations (the frontometaphyseal dysplasia-OPDS group) or a neuronal migration disorder (x-linked heterotopia) according to the portion of the molecule that is affected [6]; and FGFR2 and FGFR3 mutations may result in quite heterogeneous phenotypes combining skeletal dysplasias, syndactyly and oligodactyly, synostosis, and a variety of ectodermal findings [7]. The basis of these intriguing observations seems to be the functional topology of structural molecules and of receptors, with different functions in different domains of the protein, as well as the different partners these molecules may have in different tissues and at different times during development. Clearly, we must go beyond a simple one gene-one disorder concept and try to consider the molecular network and the temporal framework around the gene or protein involved.

## The recognition of pathways

If mutations in a single gene can produce a range of different diseases, the converse may be true as well - clinically indistinguishable forms of disease can be caused by mutations in different genes. The simplest form of this phenomenon occurs when a given molecule has more than one distinct subunit; thus, osteogenesis imperfecta can be caused by mutations in either COL1A1 or COL1A2 genes that encode for the collagen chains forming type 1 collagen, much as propionic acidemia can be caused by mutations in either PCCA or PCCB.

Further complexity is present when one phenotype is caused by mutations in genes that do not form multimeric proteins but act either synergistically or sequentially in a biological complex. Infantile Osteopetrosis can be caused by mutations in a subunit of a proton pump or in a chloride channel; mutations in carbonic anhydrase may produce yet another (milder) form. The affected biological process (or pathway) is acidification of the lacunar space delimited by attachment of the osteoclast to the bone surface, and the phenotype is similar regardless of which individual step is impaired [8,9]. Severe chondrodysplasia punctata can result from at least seven different genetic defects that involve either the biogenesis of peroxisomes, or the biosynthesis of cholesterol; it is hoped that the elucidation of the underlying pathogenetic processes will shed light on the relationship between lipid and cholesterol metabolism and calcification, a process that is ill understood so far but could reveal some target for therapeutic intervention. Non-ketotic hyperglycinemia (different components of the glycine cleavage system) or the type 1 glycogenoses (glucose 6-phosphatase and coupled transporters; GSD1a, 1b, 1c) may be cited as counterparts in metabolic disorders.

Yet another possibility is that of pathways sharing a common final step. The FGFR signaling pathway that is over-activated in achondroplasia and related disorders works via the so-called MAP-kinase pathway of intracellular signaling. One step in this pathway is regulated by cyclic GMP produced by a C-natriuretic peptide receptor with guanylate cyclase activity: this receptor is mutated in a phenotypically similar disorder, acromesomelic dysplasia. Thus, CNP acts on cartilage cells to antagonize FGFR3; indeed, in an example of "dueling pathways", an achondroplasia mouse model was rescued to normal growth by simultaneous overexpression of CNP [10]. Understanding pathways and their intersections will be of growing significance.

## Pathways as potential targets of intervention

The benefit of placing a particular disorder in a specific pathway lies in the recognition of a potential therapeutic context. The relationship between achondroplasia and acromesomelic dysplasia has been mentioned above. X-linked hypophosphatemic rickets (phosphate diabetes) is a relatively common disorder affecting both males and females, albeit greater severity is seen in males. Identification of the responsible gene, PHEX, initially posed a riddle as to the function of the encoded protease. Subsequent identification of FGF23 as the gene responsible for autosomal dominant hypophosphatemic rickets and of FGF23 overproduction in tumor-associated rickets led to the proposal that FGF23 might be the natural substrate for PHEX, and more generally to the recognition of a previously unsuspected FGF23-based phosphaturic regulation mechanism [11]. Pharmacological modulation of this mechanism is conceivable and would be of potential therapeutic benefit for hypophosphatemic ricktes. Genetic disorders have once again revealed physiological mechanisms.

Marfan syndrome is one of the more common genetic disorders. It is caused, in the vast majority of cases, by mutations in fibrillin 1, a component of elastic microfibers. While some features of Marfan syndrome such as aortic dilatation and lens luxation might be explained (albeit not simply) by weakness of connective tissue, overgrowth is difficult to explain in these terms. The observation of fibrillin acting as a biological reservoir or buffer for transforming growth factor beta (TGFβ) led to the postulation of a mechanism involving increased growth induced by reduced TGFβ binding by fibrillin [12]. This concept gained momentum when Marfan syndrome variants were found to be caused by mutations in TGFβ receptors. Finally, individuals with diaphyseal dysplasia Camurati-Engelmann type (CED) have a body habitus similar to that observed in Marfan syndrome; CED is caused by mutations in the TGFβ gene that affect the latency associated peptide function and thus produce increased TGFβ activity. Thus, the three disorders delineate a potent growth-related biological effect of TGFβ. It is tempting to speculate that modulation of this system may be responsible not only for genetic variations in body length but also in growth impairment seen so often in chronic diseases in childhood; it is an obvious potential target for therapeutic intervention.

Recognition of the role of TGFbeta signalling in the pathogenesis of Marfan syndrome has provided an unexpected bonus. The relatively old and therefore well-known drug, losartan, an AT_1 _syndrome antagonist, is able to prevent aortic dilatation in a mouse model of Marfan [13] and preliminary results in patients are encouraging [14]. Another very rare but fascinating disease, Hutchinson-Gilford progeria, may be influenced by treatment with currently available inhibitors of farnesylation [15,16]. Thus, recognition of molecular pathogenetic pathways may not only pave the way to the development of new drugs, but also identify existing drugs as therapeutic agents.

## Approaches to molecular therapy

Many of the examples cited above illustrate how "skeletal" disorders can be caused by malfunctioning of structural molecules but also of metabolic pathways or of signaling cascades: they are just examples of common molecular mechanisms. The therapeutic approaches are similarly heterogeneous. Mouse models are a crucial step for every single one. Thus, thiol replenishment to push intracellular production of sulfate is being studied in a mouse model as a potential therapy for the sulfate transporter disorders [17]. Enzyme replacement therapy has been developed for a larger number of disorders including MPS IV and hypophosphatasia. Pilot studies in patients with Morquio syndrome and with hypophosphatasia have been conducted; there are good reasons to assume that the enzyme therapy for Morquio disease, with a phenotype that arises essentially from cartilage, will be less successful than that of hypophosphatasia. The FGFR and CNP signaling pathways can be targeted by specific agonists (CNP) or by antibodies. Modulation of the FGF23 phosphaturic system may lead to treatment of hypophosphatemic rickets. Modulation of the LRP5/dickkopf system may improve bone density in the Osteoporosis-Pseudoglioma symdrome (OPGS) well as in other disorders [18]. These exciting prospects make it easier to accept the fact that gene therapy may still be years to come.

An even more promising approach derives from the fact that many key molecules have kinase activity. FGFRs are tyrosine kinases, while TGFβ receptors have serine/threonine kinase activities. Both classes of molecules have been scrutinized intensively for the identification of pharmacological inhibitors. Indeed, inhibition of the Philadelphia chromosome associated tyrosine kinase using the specific inhibitor, imatinib (Gleevec or Glivec)^®^, is highly efficient in the therapy of leukemia. Imatinib and the newer tyrosina kinase inhibitors are proving useful for the treatment of diverse disorders associated with fibrosis [19,20]. Hopefully, kinase inhibitors of sufficient specificity will be developed in the future and will be useful in the treatment of genetic disorders associated with aberrant kinase signaling.

## The potential of chaperones

Molecular therapy for disorders of skeletal growth and homeostasis may profit from a glance at the neighbouring field of metabolic pediatrics. Here, concepts such as substrate reduction, product replenishment and selective metabolic block have been developed and are commonly applied. One of the most exciting developments in recent years has been the recognition of chaperone effects. Mutant proteins are often retained within the endoplasmic reticulum by a quality control mechanism that recognizes their imperfect folding. The proteins involved in accelerating correct protein folding, in guiding the way of correctly folded proteins through the biosynthetic steps in the ER and the Golgi, and in retaining unfolded or misfolded proteins are called "chaperones". While these mechanism evolved to prevent the accumulation of misfolded proteins and are beneficial, they may actually be deleterious when it leads to the degradation of a protein that, albeit imperfect, has some residual activity - and the absence of which causes severe "monogenic" disease. The most common mutation in CFTR gene, the cystic fibrosis chloride channel, is F508; CFTR protein bearing this deletion has considerable residual activity but does not reach the cell membrane, being retained within the cells and routed for degradation because of the single missing amino acid. For the deltaF508 homozygote, delivery of the protein - albeit imperfect - to the cell surface would be more favourable than its intracellular degradation. Pharmacological interventions aimed at rescuing the mutant CFTR and allowing its delivery to the cell membrane have been successful in cell culture models, the most efficient compound being curcumin, a component of widespread and cheap curry powder [21]. It remains to be seen whether this approach is effective in vivo.

Cautious optimism is justified in view of other examples of successful chaperone therapy such as the use of galactose to enhance residual alpha-galactosidase therapy in Fabry disease or the use of a substrate analogue to enhance betagalactosidase activity in GM1 gangliosidosis [22,23]. Perhaps the most important case yet of successful chaperone therapy is the beneficial effect of tetrahydrobiopterin (BH4) administration in phenylketonuria (PKU). Initially developed to stimulate phenylalanine hydroxylase (PAH) activity in individuals who have PKU because of a metabolic defect in the synthesis or regeneration of BH4 that is a cofactor of PAH, BH4 has proven beneficial even in a significant proportion of individuals who have mutations in the PAH gene itself ("classical" PKU) [24]. Available data suggest that the mechanism increasing PAH activity is not a direct stimulation of activity, but rather a chaperone effect of BH4 leading to increased production of PAH, perhaps through a binding-induced stabilization of mutant molecules.

## From treatment of disease to molecular therapy aimed at specific mutations

Research has shown that nonsense mutations cause premature translational termination and are the reason for 5 to 70% of the individual cases of the majority of inherited diseases [25]. Nonsense-mediated mRNA decay (NMD) is an mRNA surveillance pathway which ensures the rapid degradation of mRNA containing premature translation termination codons (PTCs or nonsense codons), thereby preventing the accumulation of truncated and potentially harmful proteins. In a disease such as non-sense mediated cystic fibrosis the increase of specific protein synthesis from less than 1% to 5% may have the effect of reducing considerably the severity or even eliminating the consequences of the disease [26,27]. PTC124, a drug able to induce ribosomal readthrough of premature but not normal termination codons, can promote in human and mice cells dystrophin production, expressing dystrophin nonsense alleles [28]. This drug may have considerable scope in clinical potential for the treatment of several genetic disorders which have few or even no therapeutic options. This pioneering approach is one of the first to put personalized medicine to the test where the main focus is not on the treatment of a disease, but is rather on the treatment of a specific genetic defect.

In this way "readthrough" by means of stop codons is used for the therapy of non-sense mediated diseases; on the other hand nonsense-mediated mRNA decay can fail and be a cause of mental retardation. In fact mutations in UPF3B, a member of NMD complex, are known to give rise to syndromic and nonsyndromic mental retardation [29]. In syndromic cases the patients can have the Lujan-Fryns phenotype or the FG phenotype providing further evidence for the overlap of these two clinical conditions.

Another example of personalized molecular medicine has been investigated by van Deutekon and colleagues who chose to create a small antisense oligonucleotide that would enable the cellular machinery to "miss out" an exon in the mutated Duchenne muscular dystrophy gene by blocking its inclusion during splicing [30]. The drug is a small modified nucleic acid named PRO051 that, by blocking the inclusion of exons adjacent to DMD mutation, restores the reading frame and allows the production of a form of dystrophin with some residual function. Although the dystrophin produced under the splicing modulation with PRO051 is not normal, it probably retains considerable residual function, as shown by the condition of patients with clinically milder Becker's muscular dystrophy who have similar or identically modified dystrophins. However there are still various problems which need to be addressed with regard to this approach: firstly the optimum number and quantity of injection doses have yet to be satisfactorily established, secondly the possible toxic effects are not yet fully known and are still under investigation, thirdly the drug being used would only treat a minority of patients with DMD and other sequences will have to be researched in order to treat patients with different mutations. So it is clear that although important steps have been taken, we are still a long way from achieving personalized molecular medicine [31].

## Transplantation and the quest for the human stem cell

Bone marrow transplantation has become a therapeutic possibility for many metabolic diseases, although the efficiency and risks of the procedure itself and the actual therapeutic value of successful bone marrow engraftment are variable and still suboptimal and thus, very careful evaluation is indicated in every single case.

Contrary to what might be expected given the nature (and the name) of the procedure, the process of BMT has little or no therapeutic value in most skeletal dysplasias that affect cartilage tissue. This may have to do with the fact that cartilage is largely avascular. Initial claims of BMT effectiveness in Osteogenesis Imperfecta remain to be substantiated. BMT is highly effective in Osteopetrosis, a disease of bone marrow-derived macrophages. Hope is that further progress in the selection of stem cells and perhaps in the conditioning regimes may lead to an extension of BMT indications.

Organ transplantation is the mainstay of therapy in many pediatric renal disorders and in some liver disorders where the indication is malfunction of the organ itself. In metabolic disorders, the indication to organ transplantation may derive not only from impairment of whole organ function but also from impairment of its metabolic function only, such as in severe organic acidemias or in oxalosis. In hepatorenal tyrosinemia, liver disease results from a profound disturbance in genetic expression induced by the accumulation of toxic metabolites, and liver transplantation used to be performed frequently. While the application of a metabolic blockade with NBTC delivers dramatic clinical and prognostic amelioration, the study of liver cell behaviour in the mouse model of tyrosinemia is yielding fundamental insights into the regulation of differentiation and proliferation of hepatocytes as well as into the possibility of using bone marrow-derived cells to obtain viable and metabolically intact hepatocytes even in the absence of any stable bone marrow engraftment [32-34]. Preliminary results indicate that this may be the case even for renal tubular epithelial cells [35]. Even partial restoration of correct enzyme activity or protein production might result in clinical cure of several genetic disorders. The study of cell regeneration and cell fusion in liver and other tissues carries great promise to result in new therapeutic options for genetic disorders.

## Diseases, physicians, patients, and the pharmaceutical industry

Ever since the discovery of diseases caused by enzyme deficiencies, physicians and scientists have tried to design therapeutic strategies aimed at supplying the deficient enzyme - with plasma infusion, with cell transplantation, with bone marrow transplantation. Besides bone marrow transplantation, enzyme replacement therapy (ERT) for β-glucocerebrosidase deficiency (Gaucher disease) was the first ERT to show clinical utility. ERT for Gaucher disease has been developed at the National Institutes of Health [36] and originally licensed to the company, Genzyme, under the Orphan Drug Act. Marketing of the enzyme preparation has proven extremely successful. Almost twenty years later, more ERT products are in routine use, and others are close to their introduction to the market. Genzyme has become one of the financially most successful biotechnology company ever, its stock prices having increased by approximately 600% from 1992 to 2005 [37,38]. Conversely, national medical systems are in no better health now than then.

The existence of a strong pharmaceutical industry is of great importance, and research carried out by industry is significant and beneficial. However, in the case of Alglucerase^®^, the high price of the preparation immediately raised questions concerning public vs. private contributions in the development of that therapy [39]. Unfortunately, the hope that was originally held in recombinant DNA production technologies does not seem to have been justified, as the price of Gaucher treatment with "recombinant" enzyme is higher, not lower, than that of the placenta-derived enzyme. Thus, the questions concerning the payment for extremely expensive treatments of rare disorders raised in 1992 are still unanswered; specifically, the cost of life years, even after adjustment for quality of life, is much higher than that of all other available drugs. The long-term implications of highly expensive treatments for rare disorders remain a matter of debate [40].

Another observation worthy of reflection is that upon introduction to the market of ERT for MPS VI, the producing company distributed publicity items (brochures and bags) that explicitly questioned whether patients with MPS VI had been recognized and diagnosed. A similar strategy, though not as explicit, was used years earlier when the Gaucher ERT had been marketed in Europe: the warning that physicians might have missed the diagnosis was expressed in sponsored articles both in the medical and in the lay press. If the traditional paradigm was patients and physicians in search of a treatment, this was an inversion - a lucrative treatment in search of patients, and pressure being put on physicians not to miss them.

Similar strategies have been enacted by the growth hormone industry. More and more indications, some of which of questionable significance, have been recognized and - most importantly - been pushed through approbation by governmental agencies to obtain reimbursement by medical insurances. While growth hormone therapy for indications other than true growth hormone deficiencies may bring benefit to affected individuals, the high cost of such therapies means that significant amounts of money are diverted from other causes that are potentially of similar or even higher benefit, but for which there is no lobby; seen from inside the pediatric system, it is clear that the recognition of additional indications for GH therapy is driven more by the growth hormone industry than by pediatricians or patients. In Germany, industries producing growth hormone have formed a "consortium" with the declared aim of promoting research and dissemination of results; *de facto*, it seems clear that these industries are forming a trust to prevent competition and thus to prevent a lower pricing of GH treatment. Interestingly, advertising by these industries suggests that "short stature is not unavoidable" and thus aims at a very broad audience, not just physicians dealing with children with rare conditions.

In conclusion, a situation is developing in which the pharmaceutical industry is putting pressure on governmental agencies to grant recognition of drugs and of treatment indications, and secondarily put pressure on physicians "not to miss" patients with these treatable conditions. Often, collaboration of physicians is obtained by allocation of small financial rewards for "observation" of patients. While this is not bad *per se*, the high price of these drugs leads to a situation where treatment priorities are set by the industry and not by responsible physicians. The stronger marketing strategies of pharmaceutical industry may be favoured by the Orphan Drug Act that grants monopoly and tends to block the development of competition. We can hope that industry will continue to fulfill its role in the development and marketing of new treatments but that conditions of monopoly over individual drugs will not be the cause of financial restrictions and social inequalities.

## Roles of self-help groups and parents organizations

Patients' organizations and - in the case of pediatrics - parental organizations ("self-help groups") are emerging as the new players in the developing relationships on the health market. The primordial relationship between the patient and the physician first had to take the third partner into account - the health insurer as the payer. Meanwhile, industry is proposing itself as the fourth partner - traditionally wooing the physician in order to obtain favourable prescription of one or the other profitable drug, but recently - as in the case of ERT - with a new preposterousness.

It is not only acceptable, but also fair and desirable that individuals affected by severe and/or rare disease join forces in order to share experiences, offer mutual assistance, and act together to put forward their agenda. Potential areas of activity of patients' organisations are (among others) the exchange of information concerning treatment modalities and medical centers through meetings and brochures, the organization of cooperatives to buy or import medical or dietary supply at favourable price, the support of research, the counseling of medical researchers as to what are the objectives for clinical and basic research, the support of those research projects by helping in recruiting patient cohorts or in fundraising, and political lobbying to obtain favourable legislation. The efficiency of patients' organizations can be so high that patients may quickly obtain a degree of competence in "their" disorder ("empowerment") that the average physician may find much more difficult to obtain; thus, the knowledge advantage that used to play in favour of the physician can be inverted; this can also occur through the internet, although on the latter, information is usually much less organized and the wealth of information is not matched by quality and structured presentation. Examples are known where patients' organisations have exerted pressure on individual patients and families recommending one treatment over another and even one physician over another. Industry has recognized the growing power of such organizations, with the result that more and more sponsoring is taking place directly between industry and patients' organizations, bypassing the physicians who should decide upon the indications to treat.

How is the relationship between pediatricians and parental organizations going to evolve? Clearly, parents' organizations are here to stay; they can potentially assume a highly beneficial role in the health care system, taking on responsibility and providing assistance that the physician is unable to provide because of financial and time constraints. However, a deontological code of honour should be adopted in order to ensure that patients' (and parents') associations do maintain their own independence and at the same time allow independence of individual patients and of physicians.

## Conclusions

Aren't our times interesting? New molecular insights are flowing continuously and providing us with new concepts. Leads from these exciting results can be taken up by cell biologists and pharmacologists to develop new treatments. However, the scenario around us is moving; the partners we have to rely upon as physicians - the medical insurers and the pharmaceutical industry - are changing, a change dictated by increasing economical pressure that threatens the alliance with physicians; and the biological progress may not benefit the patients as quickly and directly as we might hope, or create new inequalities in the access to therapy. The task of developing new diagnostic procedures and effective treatments is formidable, but the task of bringing these to the market in a way that is socially sustainable is just as important. Our most important partner, the patient (for pediatricians, the sick child and its parents and advocates), is also changing; he is growing up and demanding a more mature relationship with the physician as a partner more than as a leader. In this perspective, the health players - patients and their organisations, physicians, insurers and the pharmaceutical industry - will have to agree on what can be termed "sustainable development" in a nation's health system. Pediatricians should be aware of these potential conflicts and take a proactive attitude or otherwise risk becoming more and more debased to care providers in a system that is designed and maintained by others.

## Competing interests

The authors declare that they have no competing interests.

## Authors' contributions

ASF conceived of the study; LG participated in its design and coordination. Both authors read and approved the final manuscript.
